# E-Health Interventions for Suicide Prevention

**DOI:** 10.3390/ijerph110808193

**Published:** 2014-08-12

**Authors:** Helen Christensen, Philip J. Batterham, Bridianne O’Dea

**Affiliations:** 1 School of Medicine, Black Dog Institute, University of New South Wales, Sydney, NSW, 2031, Australia; 2 Centre for Mental Health Research, The Australian National University, Canberra, ACT, 0200, Australia; E-Mail: philip.batterham@anu.edu.au; 3 School of Medicine, Black Dog Institute, University of New South Wales, Sydney, NSW, 2031, Australia; E-Mail: b.odea@blackdog.org.au

**Keywords:** internet, suicide prevention, e-health, social media, screening

## Abstract

Many people at risk of suicide do not seek help before an attempt, and do not remain connected to health services following an attempt. E-health interventions are now being considered as a means to identify at-risk individuals, offer self-help through web interventions or to deliver proactive interventions in response to individuals’ posts on social media. In this article, we examine research studies which focus on these three aspects of suicide and the internet: the use of online screening for suicide, the effectiveness of e-health interventions aimed to manage suicidal thoughts, and newer studies which aim to proactively intervene when individuals at risk of suicide are identified by their social media postings. We conclude that online screening may have a role, although there is a need for additional robust controlled research to establish whether suicide screening can effectively reduce suicide-related outcomes, and in what settings online screening might be most effective. The effectiveness of Internet interventions may be increased if these interventions are designed to specifically target suicidal thoughts, rather than associated conditions such as depression. The evidence for the use of intervention practices using social media is possible, although validity, feasibility and implementation remains highly uncertain.

## 1. Introduction

E-health interventions for suicide prevention can be classed into three categories. First, the Internet can be used to help individuals self-screen: to identify whether they might be at risk for suicide or a mental health problem. Through screening and feedback, it may be possible to increase service use, by directing at-risk individuals who would not otherwise seek help to access appropriate evidence-based online programs or to access traditional mental health services [1]. Second, web applications, both guided and unguided, have been developed to provide psychological interventions to assist in reducing suicidal behaviour and lowering suicidal ideation. Guided interventions involve a therapist or a researcher assisting the user through the program either through email or over the telephone, whereas unguided are self-help, automated programs which can be initiated and used directly by the public. The third type of intervention is one where a person is considered to be at risk of suicide because of the nature of their social media use. Here, tweets, status updates, comments or posts indicative of suicide ideation are used to classify those at risk. Such content can be identified in real-time by other users or by computerised language processing and progress in this area has accelerated. The aims of the present paper are to review the evidence around these three styles of intervention. Three separate literature reviews were conducted. The first examined the evidence for whether online screening for suicide might be effective in reducing suicidal ideation and behaviours. The second reviewed web interventions, updating two recent reviews of web based suicide prevention [2,3] and distinguishing two approaches: web interventions that target suicidal behaviour and using depression therapies; and interventions that target suicidal behaviour using suicide-specific therapies. The final review examined the use of social media platforms for identifying those who may be at risk of suicide.

## 2. Methods

Comprehensive literature searches for all three reviews were conducted in March 2014 using the Medline, PsychInfo, and Cochrane Library databases. Conference abstracts, non-peer reviewed papers, non-English language papers, and PhD theses were excluded from all three reviews. All articles were screened for eligibility by two independent researchers. The specifics of each search are outlined below.

### 2.1. Identifying Screening Programs

Search terms indicative of internet technology (*computer or computer-based or cyber or cyberspace or electronic or* “*electronic mail*” *or email or e-mail or internet or internet-based or net or online or virtual or web or web-based or web-based or* “*world wide web*” *or www or* “*social media*” *or* “*social network*” *or blog or forum*), screening (screen* or assess*) and suicide (suicid*) were employed. The search resulted in 855 articles, of which 98 were excluded due to being in a non-English language or animal-based and a further 162 were excluded as duplicates. The remaining 595 abstracts were screened for relevance, with 78 papers identified as potentially meeting the criterion of reporting on the screening of individuals for suicidal ideation or behaviours using the internet. Full-text copies of these papers were then obtained for further scrutiny and reference checks conducted to identify papers that may have been missed in the search. After examining the full text of these articles, 47 did not meet the criteria of the screening review. Hierarchical reasons for exclusion were not being a peer-reviewed paper (11 papers), not using online data collection (20 papers), not including a measure of the respondent’s suicidality (eight papers), not reporting on suicidality outcomes (seven papers), or being a review paper (one paper). One additional relevant paper was identified in reference checking, resulting in 32 papers from 30 studies that reported on online assessment of suicidal ideation or behaviours.

### 2.2. Identifying Web Programs

In addition to the databases stated above, the Centre for Research Excellence of Suicide Prevention (CRESP) Suicide Prevention Database (http://cresp.edu.au/databases/sprct) was also used for this review. Search terms indicative of suicide, self-harm and mobile or online applications were employed: “Self harm OR self-harm OR deliberate self-harm OR deliberate self poisoning OR self cutting OR self-inflicted wounds OR deliberate self cutting OR suicid* OR suicide gesture OR suicidal behavio* OR suicidal ideation OR suicide attempt OR self-mutilation OR auto-mutilation OR auto mutilation OR self-injury OR self-injurious behavio* OR self destructive behavio* OR self-poisoning and overdose OR drug overdose” in Title, Abstract, Keywords and “*trials OR randomised controlled trials OR randomized controlled trials OR meta-analysis*” in Title, Abstract, Keywords and “*internet OR mobile OR web OR email OR e-mail OR online*” in Title, Abstract, Keywords.

The search yielded a total of 198 abstracts which was reduced to 109 after the removal of duplicates. Of these, nine papers met inclusion criteria. Inclusion and exclusion criteria were applied in order to identify any internet or mobile-based interventions that included a measure of suicidal behaviour. The trials did not need to explicitly target those experiencing suicidal behaviours, but they were required to measure participants’ level of suicidality prior to program commencement and following program completion. Studies examining non-suicidal self-injury were not included. Due to the low numbers of trials, studies without control or comparison groups were included in addition to trials including control groups. The control group could consist of a wait-list, treatment-as-usual, or another treatment. There was no restriction on participant age. 

Studies were excluded if they did not include an intervention, if suicidality was not measured as a primary or secondary outcome, and if the intervention was not internet or mobile based. One paper was excluded as the research design and sample was identical to another paper written by the same authors. Further, the study led by Merry [4] employed the Kazdin Hopelessness Scale in place of a suicidal behaviour measure. Considering this scale is widely used as a proxy for suicidal ideation, the study was included. 

Two of the studies in the review are not discussed further. These are: Marasinghe, *et al.* [5]; and Wagner, *et al.* [6]. The first was eliminated because it did not involve a web component. The second because it was difficult to discern the type of therapy, and the extent to which the intervention was delivered online (it was not clear whether both groups received a paper and pencil manual).

### 2.3. Identifying Social Media Interventions

Search terms indicative of suicide, self-harm and social media were employed: suicid* OR suicide gesture OR suicidal behavio* OR suicidal idea* OR suicide attempt OR self-mutilation OR self harm OR self-harm OR deliberate self-harm OR deliberate self poisoning OR self cutting OR self-inflicted wound OR deliberate self cutting AND social media OR internet OR web OR online OR blog* OR online social network* OR website OR twitter OR Myspace OR Facebook OR social networking site* OR bebo OR tweet* OR status update OR post*AND screen* OR assess OR prevent* OR track*. Using Kaplan and Haenlein’s [7] classification of social media, the current review focused explicitly on blogs/microblogs (e.g., Twitter), and social networking sites (e.g., Facebook, Myspace, Bebo). Content communities (e.g., YouTube), virtual worlds (e.g., Second life), MMORGs (e.g., World of Warcraft) and collaborative projects (e.g., Pinterest) were excluded. Articles that related to online discussion forums, online support groups or internet message boards were also excluded. The search yielded 1934 following number of articles. Of these, only 13 papers met inclusion criteria. Papers published prior to 2000 (the onset of social media) were excluded alongside those that focused on non-suicidal self-injury, murder-suicides or mental illnesses such as depression, which may increase the risk of suicide. A search for similar articles was conducted on all relevant papers; however, this returned zero results. Citation lists for each included article was also screened for other related papers which yielded an additional three papers. The article titled “Mining Twitter for Suicide Prevention” [8] is not discussed further as the publication source could not be identified. A total of 15 papers were included.

## 3. Results

### 3.1. Online Screening for Suicide Ideation

Of the 32 papers that met inclusion criteria, only six (19%) had direct relevance to online screening programs. The details of these papers are provided in Table 1. Of the remaining papers not detailed in the table, 23 (72%) focused on assessing risk factors or prevalence of suicidal ideation or behaviours using cross-sectional web surveys, while one paper reported on the cost-effectiveness of an online suicide prevention trial, one paper reported on the psychometric properties of an online behavioural health measure and another reported on a cross-sectional survey assessing reasons why people seek help for suicide online. All of the six papers on screening were based on studies from the United States. Five of the papers focused on young people, including four on university students and/or staff. Sample sizes ranged from 374–13155 (*M*: 2916, Median: 1010) and 45% of all participants were female. Most studies used the PHQ-9 [9] to assess suicidal ideation, usually in association with a lifetime suicide attempt item. The remainder used binary prevalence measures (*i.e.*, presence/absence of suicidal ideation, suicide plan, or suicide attempt).

Three of the papers [10,11,12] reported on the use of an online screening program developed by the American Foundation for Suicide Prevention in the university setting. Overall, these studies reported that online screening was effective for identifying students with history of suicidal ideation or behaviours. The direct referral of at-risk students to in-person services, such as campus counsellors, was also found to be effective, although only a minority (ranging approximately 10%–20%) of at-risk students agreed to service referral and fewer received services. Another study [13] examined web-based screening among adolescents presenting at the emergency department of a children’s hospital, finding that 65% of adolescents agreed to screening, resulting in a 70% increase in identification of psychiatric problems and a 47% increase in assessments by a social worker or psychiatrist. Lawrence, *et al.* [14] tested the feasibility of using the PHQ-9 within a web-based screener administered in outpatient clinics to screen for depression and suicidality among people with HIV. In this program, 14% reported some level of suicidal ideation and 3% were deemed high-risk and referred to services. Critically though, none of the identified studies included a control (non-screened) group for comparison, so they were unable to assess whether screening and referral alone was sufficient to increase help seeking or to improve mental health outcomes.

**Table 1:** Studies of online screening for suicidal thoughts or behaviours identified in the review.

Paper	Topic	N	% Female	Location	Population	Measure
Fein, *et al.* [13]	Evaluation of emergency department psychiatric screener	857	56	USA	Adolescents; Emergency dept	Behavioural Health Screener
Garlow, *et al.* [10]	Description of a university screening program	729	72	USA	University students	PHQ-9 + past attempts
Haas, *et al.* [11]	Description of a university screening program	1162	70	USA	University students	PHQ-9 + past attempts
Lawrence, *et al.* [14]	Description of a suicide screening program	1216	21	USA	People with HIV; primary care	PHQ-9
Moutier, *et al.* [12]	Description of suicide/depression screening program	374	--	USA	University staff & students	PHQ-9 + past attempts
Whitlock, *et al.* [15]	Responses to being asked about suicide, self-harm	13,155	43	USA	University students	National Comorbidity Survey items

One additional study examined the acceptability and risk of online screening for suicidal ideation and behaviours, also among university students [15]. This study concluded that such screening was generally acceptable, with few individuals (<3%) reporting negative experiences as a result of the screening. Individuals with previous suicidality had greater discomfort with the survey but such individuals also found it more thought-provoking than those without previous suicidality [15].

### 3.2. Web Applications for Suicide Prevention

Outlined in Table 2, six studies examined suicide outcomes arising from the use of a web intervention which targeted depression. Of these, two studies used adolescent samples. A pre-post study (*n* = 83) of general practice adolescent patients with suicidal ideation (but not frequent ideation or actual intent) found reductions in self-harm thoughts and depressive symptoms at 6 weeks and 12 weeks using the “PROJECT CATCH-IT” web program (CBT, IPT and parent workbook) [16]. A non-inferiority RCT (*n* = 94) of psychiatric outpatients with depression compared a computerised self-help program “SPARX” (CBT) with TAU (face to face therapy) and found that SPARX was non-inferior on levels of hopelessness (a proxy measure of suicide ideation) [4]. Four studies targeted adults. Two Australian studies examined changes in suicide ideation using a pre-post design. In the first, 299 general practice patients with suicidal ideation undertook an internet intervention for depression (CBT, homework and clinician contact) and the researchers found a reduction in suicidal ideation [17]. A pre-post study (*n* = 359) of depressed or suicidal general practice patients, evaluated the “Sadness Program” (internet based CBT, homework, supplementary resources) and found reduction in suicidal ideation [18]. A third study (*n* = 105) of depressed patients with suicidal thoughts employed a RCT methodology. The researchers compared “Deprexis” (online CBT for depression) with wait list controls and found decreased scores on depression, dysfunctional attitudes and improved quality of life, but no difference on either suicidal thoughts and behaviour [19]. A second RCT (four arms) (*n* = 155) of depressed callers to Lifeline compared web-based CBT, web-based CBT plus telephone call, telephone call back line and TAU found no differences in the rate at which suicidal thoughts dissipated between the four conditions [20].

Taken together these findings from both the adult and the adolescent studies show that suicide ideation drops over time in response to internet interventions (the Deprexis results is the only anomaly). The two RCT trials [19,20] also demonstrate that depression websites have specific effects on depression symptoms above those of the control conditions. However, the studies were not able to establish that suicide ideation falls as a function of the depression CBT provided by the interventions. In other words, suicide ideation seems to drop in both depression and control conditions. These findings do not rule out the possibility that websites which target specific characteristics of suicidal thoughts and behaviour such as rumination disruption or mindfulness may be more effective than either depression interventions or the “passage of time”. There is only one RCT that has been published which focuses on a specific intervention for suicidal thoughts. This study, compared an online self-help program (6 modules of CBT with DBT, PST, MBCT as well as weekly assignments and automated motivational emails) with a waitlist control group and found reductions in suicidal thoughts and levels of hopelessness in favour of the self-help program [21] and improved cost effectiveness [22].

### 3.3. Social Media for Suicide Prevention

Table 3 outlines the relevant papers and associated findings. Of the 15 articles identified, six were classified as case studies examining either one individual’s social media use (*n* = 5), or, the social media use of a single organisation (*n* = 1). In the single organisation case study, Boyce [23] described the social media use of a suicide prevention agency (“Samaritans”) in the United States (U.S.). This case study provided brief recommendations for the future but did not include any data in regards to the reach, impact or effectiveness of such social media use. The remaining five individual case studies focused on young males aged between 13–29 years living in either China or the U.S., or location undisclosed. In 2011, Ruder and colleagues [24] presented commentary on a case involving a suicide note posted on Facebook by a 28 year old male who died by suicide. No data or social network analysis was conducted. Lehavot, Ben-Zeev and Neville [25] outlined the ethical considerations involved with a therapist using Facebook as a monitoring tool for postings of suicidal imagery in a male patient. No data or social network analysis was conducted. Ahuja, *et al.* [26] briefly discussed the suicidal postings of a male in his late twenties with a history of mental illness and the potential for offline social network intervention. No data or social network analysis was conducted. Using sentiment analysis software, Fu, Cheng, Wong and Yip [27] examined the reactions and patterns of information diffusion to a self-harm post made by a male using the Chinese social networking site “Sina Weibo”. Li, Chau, and Wong [28] examined the relationship between blog posting intensity and language use to explore the suicidal processes of a 13 year old male. The specific blog site was not identified.

Four of the overall papers, including one brief correspondence, were non-systematic literature reviews discussing the use of social media for suicide prevention. Two of these reviews, published by Luxton and colleagues [29,30] described the social media platforms currently being used, or those with potential, for suicide prevention. The most recent review by Luxton, June and Fairall [29] found a total of 580 Twitter groups and 385 blog profiles on blogger.com designated to suicide prevention, one social networking site designed for social media prevention in the US (lifeline-gallery.org), and discussed the application of internal functions that could act as alert systems for potential suicide behavior. Luxton, *et al.* [29] also discussed the presence of suicide notes on social media, but did not refer to any data or particular studies. The third review [31] outlined the potential benefits and complications of social networking sites as a therapeutic intervention for self-injury but did not include any data or references to a specific platform. The brief correspondence [32] presented policy initiatives and internal functions of social media that could be adopted for suicide prevention.

Only five papers specifically examined the utilisation of social media for the tracking of suicide risk. These studies focused on either Twitter (*n* = 1), Myspace (*n* = 2), Facebook (*n* = 1) or other blog site (*n* = 1, Naver blog, Korea). The studies focusing on Myspace included self-reported adolescent samples with ages ranging between 13–24 years. Age range of participants in the other papers could not be determined. To our knowledge, the first study published in this area was an exploration of public Myspace blogs of New Zealand youth using automated sentiment analysis [33]. Cash and colleagues [34] further explored the content of suicidal statements made on the public Myspace profiles of adolescents aged between 13–24 years old. Using Twitter, Jashinsky, *et al.* [35] identified a significant association between suicide risk factors within Twitter and geographic specific suicide rates in the US. Using automated sentiment analysis software, Won, Myung, Song, Lee, *et al.* [36] examined whether two blog sentiments (suicide-related and dysphoria-related) along with traditional social, economic and meteorological variables significantly predicted suicide rates in Korea over a three year period (2008–2010). The final study [37] was a thematic content analysis of the portrayal of suicide and self-harm within a Facebook group in April 2009. Computerised sentiment analysis was not used.

**Table 2:** Internet and mobile programs designed to assist those experiencing suicidal ideation or deliberate self-harm.

Paper	Country & Period of Trial	Target Group (n), Age, %, Male	Research Design	Intervention Component/s	Setting	Suicide Behavior: Baseline Suicide Levels	Suicide Outcome Measure	Results
Christensen, *et al.* [20]	Australia; July 2007 to January 2009	Depressed participants who have called Lifeline (score of more than 22 on the K10), *n* = 155 (Internet only (*n* = 38); Internet + call back (*n* = 45); Telephone call back only (*n* = 37); TAU (*n* = 35)); mean age = 41.49; 18.1% male.	RCT; four arms: (1) Web-based CBT intervention; (2) Web-based CBT intervention + telephone call back; (3) proactive call back telephone line; (4) TAU. Participants assessed at pre and post intervention, and 6 and 12 month follow-up.	6 weeks of any 3 intervention conditions. Web based CBT condition (1) consisted of psycho-education provided by BluePages, and MoodGYM-interactive web application, based on CBT-5 modules. Condition 2 also included weekly 10 min call from a Lifeline counsellor	Callers to Lifeline (telephone counselling service for people experiencing crisis.)	Suicidal ideation (excluded if acutely suicidal); Mean GHQ suicidal ideation score = 1.73	GHQ-28 (4-items pertain to suicidal ideation component).	Significant reduction in suicidal ideation at post for internet only (*p* = 0.05), telephone call back only, *p* = 0.003, and TAU (*p* = 0.005); at 6-month follow-up for internet only (*p* = 0.016, and telephone call back only (*p* = 0.029); at 12-month follow-up for internet only (*p* < 0.001), internet + call back (*p* < 0.001), and telephone call back only (*p* = 0.011).
Marasinghe, *et al.* [5]	Colombo, Sri Lanka; no dates given	Patients undergoing treatment post-suicide attempt, mean age intervention (*n* = 34) = 32 years; control (*n* = 34) = 30 years, 50% male in both conditions	Single-blinded RCT—clinical trial *vs.* wait list control with post and 6 month follow-up	Clinical trials, Phase 1: 220–380 min face-to-face component; Phase 2: Brief weekly phone calls/SMS to participants for 26 weeks; Control: Usual Care followed by Phase 2 component.	Outpatient, following primary care	Recently attempted suicide, displaying suicidal intent; Mean BSSI score for control males (21.3), females (22.2), intervention males (26.7), females (25.5)	BSSI; Primary	BSSI scores—Intervention baseline to 6-months to 12-months (26.1–3.65–3.6); Control baseline to 6-months to 12 months (21.75–7.55–3.75); no *p*-value reported.
Merry, *et al.* [4]	New Zealand; May 2009 to July 2010 + follow-up in December 2010	12–19 years olds with mild to moderate symptoms of depression; mean age online Intervention (*n* = 94) = 15.55, TAU (n = 93) = 15.58	Randomised controlled non-inferiority trial—Online intervention *vs.* TAU (face-to-face therapy). Pre-post + follow-up	7 CBT-based interactive modules to be completed in 4–7 weeks	Outpatient, had sought help for depression	Indirect—depression severity (excluded those deemed high risk of suicide or self harm) ; ITT participants mean score on hopelessness for control (6.15) and intervention (6.17)	Indirect—Kazdin Hopelessness scale for children	Per protocol improvements in hopelessness were significantly greater for participants in the online intervention. ITT improvements were non-significantly larger than TAU.
Moritz, *et al.* [19]	Hamburg. Germany (online recruitment); no dates given	Participants with elevated depression symptoms; mean age intervention (*n* = 105) = 38.0 years, 22.9% male, control (*n* = 105) = 39.13, 20% male	RCT; Online self-help program *vs.* Wait list control; pre-post treatment survey (after 8 weeks)	Online self-help program for depression (Deprexis); 10 modules, CBT based	Online setting	Suicidal thoughts and behaviour (excluded patients with strong suicidal ideas); mean SBQ-R score 12.28 (wait-list controls); 11.37 (intervention)	SBQ-R, assesses suicidal thoughts and behaviour; Secondary	Significant symptom decline on depression, dysfunctional attitudes, improvement in quality of life and self-esteem. No significant improvement on SBQ-R scores.
Van Spijker, *et al.* [21]	Netherlands (Online recruitment); October 2009 to November 2010	Mild to moderate suicidal thoughts (scores between 1 and 26 on the BSSI); mean age intervention (*n* = 116) = 40.46 years; control (*n* = 120) = 41.39, 33.9% male.	RCT intervention group *vs.* waitlist control	6 modules (30 min per day over 6 weeks) of CBT with DBT, PST, MBCT + weekly assignments and optional exercises with up to 6 automated motivational emails	General public recruited via online and newspaper advertisements	Mild to moderate suicidal thoughts; BSSI mean score of 14.5 (control) and 15.2 (intervention), 16.8% had attempted suicide once and 24.1 had multiple attempts	BSSI; Primary;	Significant reduction in suicidal thoughts for intervention group compared to control group (*p* = 0.036). Non-significant reductions in depressive symptoms
Van Voorhees, *et al.* [16]	United States of America; February 2007 to November 2007	Primary Care adolescent patients, (*n* = 83), mean age = 17.39 years, 43% male	Pre-post (at 6 and 12 weeks), no control	14 modules based on CBT, IPT, community resiliency concept model (CATCH-IT); Additional parent workbook to support adolescents progress	Outpatient, following primary care	Self-harm risk (suicidal ideation) (excluded patients who expressed frequent suicidal ideation or actual intent);13% thought about suicide in past 2 weeks, 7% with serious suicidal thoughts in last month, 16% with any suicidal thoughts	PHQ-A—self-harm risk; Secondary	Significant reduction in self-harm thoughts at 6-weeks (*p* = 0.04) and 12-weeks (*p* = 0.02) and depressive symptoms at 6-weeks (*p* < 0.001) and 12-weeks (*p* < 0.001) and depressive disorder for major depression at 12-weeks (*p* = 0.047).
Wagner, *et al.* [6]	Zurich, Switzerland; November 2008 to February 2010.	People experiencing depression (score of at least 12 on the BDI-II); mean age online (*n* = 32) = 37.25 22% male, face-to-face (*n* = 30) = 38.73; 50% male.	Randomised Controlled Non-inferiority Trial; pre-post; Internet intervention *vs.* face-to-face CBT intervention	Internet based CBT intervention including structured writing assignments with individualized therapist feedback; 8 weeks	General public recruited via online and newspaper advertisements	Suicidal ideation (excluded if high risk of suicide); BSI = 3.24 (online); = 4.87 (face-to-face).	BSI; Secondary	No between group differences for any pre-post treatment measurements. Significant pre-post reduction in suicidal ideation (*p* < 0.05) for face-to-face treatment group, but not for iCBT group (*p* = 0.24).
Watts, *et al.* [17]	Sydney, Australia; April 2009 to May 2011	Primary Care patients (n = 299), mean age = 43 years, 44% male	Clinical audit; pre-post, no control	6 CBT-based lessons + homework with clinician making contact at least twice during the course	Outpatient, following primary care	Suicidal ideation (excluded “actively suicidal” patients); 54% mild, 30% moderate, 15% severe, 9% ex. severe	PHQ-9 using Q9 as measure of frequency of suicidal ideation;Primary	Significant reduction in suicidal ideation scores (*p* < 0.001) and depression scores (*p* < 0.0001).
Williams, *et al.* [18]	Australia; October 2010 to November 2011; 54% of participants from rural or remote community	Primary care patients enrolled in the Sadness Program, who were either severely depressed and/or expressing suicidal ideation, (*n* = 359), mean age = 41.59; 41% male	Quality assurance study; pre-post, no control	iCBT- The Sadness Program: 6 online lessons within 10 weeks; regular homework assignments, access to supplementary resources	Outpatient, following primary care	Suicidal ideation; PHQ9 scores = (17% severe, 8% very severe). 53% (*n* = 189) endorsed suicidal thoughts during the 2-week time period prior to commencing the program	PHQ-9 Suicide item; Primary	Significant reductions in suicidal ideation for Ss experiencing suicidal ideation (*p* = 0.001) and for Ss experiencing severe depression and suicidal ideation (*p* < 0 001). 54% of patients who completed all 6 lessons evidenced clinically significant change in depression.

CBT—Cognitive Behavioural Therapy; PHQ-9—Patient Health Questionnaire—9 item; RCT—Randomised Controlled Trial; SBQ-R—Suicide Behaviors Questionnaire-Revised; PHQ-A—Patient Health Questionnaire-Adolescent; IPT—Interpersonal Psychotherapy; iCBT—internet-based Cognitive Behavioural Therapy; SMS—Short Message Service; BSSI—Beck Scale for Suicidal Ideation; BDI-II—Beck Depression Inventory-Revised; DBT—Dialectical Behaviour Therapy; PST—Problem Solving Therapy; MBCT—Mindfulness Based Cognitive Therapy; K10—Kessler’s Psychological Distress Scale-10 items; TAU—Treatment As Usual; GHQ-28—General Health Questionnaire-28-item; ITT—Intention To Treat.

**Table 3:** Articles related to social media for suicide prevention.

Type	Paper	Design/Methods	Sample, Location & Platform	Findings
**Case studies**	Boyce [23]	Descriptive commentary	Samaritans U.S. Facebook page. *Time*: not relevant. *Data*: nil.	Argued that social media behavior can help determine the path that suicidal people take online.
	Ruder, *et al.* [24]	Descriptive commentary	A suicide note posted on Facebook by a 28 year old male who died by suicide. *Time*: not reported. *Data*: nil.	Suicide notes posted via social media may allow for timely suicide intervention by alerting other network users immediately, although understanding the relationship between online suicide notes and copycat suicides is important to consider.
	Lehavot, *et al.* [25]	Descriptive case study	Male, late 20’s, history of mental illness, location unknown, posted suicidal imagery on his Facebook profile. *Time*: not reported. *Data*: nil.	Several ethical issues, including beneficence and maleficence; privacy and confidentiality; multiple relationships; clinical judgement; and informed consent, were discussed.
	Fu, *et al.* [27]	Quantitative content analysis	A self-harm post made by a male on the social networking site Sina Weibo. *Time*: March 2011. *Data*: 5971 microblog responses were included.	Responses were classified as caring (37%), negative (23%), shocked (20%) or unemotional reposts (20%). Significant clustering was identified in the repost network in which the speed of diffusion was faster when compared to the random network.
	Li, *et al.* [28]	Computerised language processing	Male, 13 years old, located in China. Microblog site unidentified. *Time*: not reported. *Data*: 193 blog entries made in the year preceding the participant’s suicide were analysed.	The ratio of positive to negative emotion words was associated with greater posting trend. There was greater use of negative emotion over time. Progressive self-referencing appeared to be a predictive sign of suicide, although, the comparison did not show other clearly consistent patterns.
	Ahuja, *et al.* [26]	Descriptive Case Study	Male, late 20’s, history of mental illness, location unknown, posted suicidal ideation his Facebook profile. *Time*: not reported. *Data*: 3 posts taken from Facebook page.	General discussion of how social media can assist in screening for suicidality as well as preventative methods when individuals display suicidal thoughts via social media.
**Reviews**	Luxton, *et al.* [30]	Non-systematic literature review	NA	Social media provides opportunities for effective outreach and suicide prevention but cannot replace careful clinical case management. Further evaluation necessary.
	Messina & Iwasaki [31]	Non-systematic literature review	NA	A discussion of the internet uses associated with self-injury. No reference to particular social media platforms.
	Luxton, *et al.* [29]	Non-systematic literature review	NA	Social media has the potential to be used for suicide prevention within a public health framework although more research is needed on the degree and extent of the influence of social media for such purposes.
	Cheng, *et al.* [32]	Brief Correspondence	NA	Suggested that social networking sites could help prevent suicides by deleting pro-suicide groups re and automatically delivering private messages to those at risk.
**Sentiment Research**	Huang, *et al.* [33]	Computerised sentiment analysis with manual inspection	*Participants*: 15,000 Myspace users aged 15–24 living in New Zealand. *Time*: not reported. *Data*: 4273 unique blogs were examined.	Overall, 3.7% and 5% of active bloggers were potentially suicidal: 35% were identified as positive hits. 638 users out of the 4273 received a score of 1 or higher indicating that at least one match was found with the dictionary phrases. Using the exact phrases, 612 bloggers received a score of 1 or higher. Although the ability to definitively identify bloggers with suicidal tendencies is limited, the study demonstrates that computerised data mining can be used to identify users at potential risk.
	Zdanow & Wright [37]	Thematic content analysis of user statements	*Participants*: Facebook users, presumed to be teenagers. *Time*: 27 April 2009. *Data*: Group 1: 15,201 members, Group 2: 228 members.	Themes identified: normalization, nihilism, glorification, ‘us *vs.* them’, acceptance, reason, mockery. Facebook groups were found to encourage and promote positive perceptions of suicidal behavior.
	Cash, *et al.* [34]	Computerised sentiment analysis with manual inspection	*Participants*: Myspace users located in the United States, public profile, not self-identified as musicians, comedians or movie makers, had between 2–1000 friends. *Time*: 3–4 March 2008 and downloaded again in December 2008. Sample was reduced in 4 stages. *Data*: 1762 comments collected: 1038 met criteria, reduced to 490 comments. 105 comments mentioned suicide but referred to the suicide of another. Final coding revealed 64 comments related to a serious comment made by the commenter about potential suicidality.	Researchers were able to categorise ‘at-risk of suicide’ bloggers with up to 35% success and demonstrated a 14% automated identification rate. Many of these posts were related to a breakdown in personal relationships (42.2%) with some references to mental health problems (6.3%); however, for the most part, context of the statement could not be established.
	Jashinsky, *et al.* [35]	Computerised sentiment analysis with manual inspection	*Participants*: Twitter users located in the U.S. *Time*: 15 May 2012–13 August 2012. *Data*: 1,659,274 tweets from 1,208,809 users over a 3 month period. Exclusion criteria resulted in 733,011 tweets from 594,776 users: 37,717 identified as suicidal. A specific state location could be identified for 37,717 tweets from 28,088 users.	A total of 2.3% (*n* = 37,717) of users were identified as at risk for suicide. A strong correlation was observed between state Twitter-derived data for suicide and actual state age-adjusted suicide data.
	Won, *et al.* [36]	Computerised sentiment analysis comparing national, economic and meteorological data with blog posts	*Participants*: Korean microbloggers using Naver Blog. *Time*: 1 January 2008–31 December 2010. *Data*: 153,107,350 posts on 5,093,832 blogs collected over three years.	Both sentiments were associated with suicide frequency. The suicide sentiment displayed high variability and were found to be reactive to celebrity suicide events, while the dysphoria sentiment showed longer, secular trends with lower variability. In the final multivariate model, the two sentiments displaced consumer price index and unemployment rate as significant predictors of suicide.

## 4. Discussion

To date, there exists no controlled study testing whether online suicide screening can effectively increase levels of help seeking or reduce suicidal ideation or behaviours. Online screening for suicidal thoughts and behaviours appears to be acceptable among young people [15], supporting previous findings from a randomised controlled trial that screening for suicide risk using paper surveys does not increase the risk of suicidal thoughts or behaviours [38,39]. Although there is some evidence that screening is a feasible way to increase identification of suicidality and increase service referrals, research of online screening programs has not rigorously evaluated whether screening programs are effective compared to not screening. Furthermore, most of the programs reported in the literature have been conducted in selective populations, specifically, among university students in the United States. There is some evidence from paper-and-pencil assessments that suicide screening programs can be effective in specific settings with the availability of appropriate referral sources, with most of this research also conducted among young people (e.g., [40,41]). However, there is a need for additional robust controlled research to establish whether suicide screening can effectively reduce suicide-related outcomes, and in what settings screening might be effective [42,43,44]. None of the identified studies had a control condition, so they were unable to robustly assess whether online screening directly increased help seeking or led to improved mental health outcomes. This evidence gap appears to be especially conspicuous for online screening.

With respect to online suicide prevention programs, there is some evidence to suggest that suicide interventions via the web may be effective, but only if they specifically target suicidal content, rather than the associated symptoms of depression through CBT. There is no evidence CBT web-based programs do harm, so excluding those with suicide ideation from participation does not seem warranted for online programs in general. Further research targeting specific suicidal content on the web is warranted.

Although limited, there is evidence to suggest that social media platforms can be used to identify individuals or geographical areas at risk of suicide. The few studies conducted in this area have demonstrated that it is possible to use computerised sentiment analysis and data mining to identify users at risk of suicide; however, these studies have been conducted in publically available networks. Little is known about private online social networks such as those within Facebook. Furthermore, the prevalence and response rate of suicide notes posted on social media is yet to be clearly understood. The repost networks within social media may be a potential preventative method that could be activated quickly for effective intervention in emergency situations; although, current methodologies cannot fully disentangle whether suicidal postings always receive a response and if so, the nature of such responses. It is unknown if sharing thoughts and feelings this way is beneficial to an individual or whether they are placed at further risk. While evidence suggests that social media may be a viable tool for real-time monitoring of suicide risk on a large scale, future studies are needed to further validate this method, in addition to determining the underlying mechanisms for providing support via these channels.

## 5. Conclusions

This review highlights that there is currently limited evidence for the effectiveness of e-health interventions for suicide prevention. Whilst feasible, the reliability and preventative capacity of online screening for suicidality is yet to be clearly determined. Research in this area is incomplete. Furthermore, online therapeutic interventions do not appear to be effective unless content is targeted to suicidal thoughts and behavior; however, addressing risk factors, such as depression, through online CBT does not appear to cause harm. Lastly, social media shows significant potential for identifying those at risk of suicide in addition to mapping suicide contagion, although further validation research in this area is also needed.
